# Identification and Validation of Hub Genes Associated With Periodontitis and Systemic Lupus Erythematosus

**DOI:** 10.1002/iid3.70588

**Published:** 2026-11-05

**Authors:** Yafang Li, Xin Xu, Zhijing Wu, Feng Li, Jie Zhang

**Affiliations:** ^1^ Jiangxi Provincial Key Laboratory of Oral Diseases, Department of Stomatology The First Affiliated Hospital Nanchang China; ^2^ Medical College Nanchang University Nanchang China

## Abstract

**Background:**

Accumulating evidence suggests a close relationship between periodontitis and systemic lupus erythematosus (SLE); however, the shared molecular mechanisms underlying this association remain unclear. This study aimed to identify candidate genes and molecular features shared between periodontitis and SLE through integrative bioinformatics analysis and clinical validation.

**Methods:**

Gene expression profiles were obtained from the Gene Expression Omnibus (GEO) database, and differentially expressed genes (DEGs) shared between periodontitis and SLE were identified. Gene Ontology (GO) and Kyoto Encyclopedia of Genes and Genomes (KEGG) enrichment analyses were performed, followed by protein–protein interaction (PPI) network construction and hub gene screening. The expression patterns of the hub genes were evaluated in independent validation datasets, and their discriminatory performance was assessed by receiver operating characteristic (ROC) curve analysis. Immune cell infiltration and its correlations with hub gene expression were further investigated. Transcription factor–microRNA regulatory networks and candidate compounds were computationally predicted. Finally, quantitative real‐time PCR (qPCR) was performed to validate hub gene expression in clinical samples.

**Results:**

A total of 124 overlapping DEGs were identified, from which five hub genes—CCL3, LY96, FOS, MMP9, and PTGS2—were selected. GO and KEGG enrichment analyses indicated that the overlapping DEGs were predominantly associated with inflammatory and immune‐related biological processes and signaling pathways. All five hub genes showed consistent expression patterns in the independent periodontitis validation dataset, whereas FOS, LY96, and PTGS2, but not CCL3 and MMP9, showed consistent differential expression in the independent SLE validation dataset. ROC analyses demonstrated varying discriminatory performance across the analyzed datasets. Immune infiltration analysis revealed differences in estimated immune cell profiles in both disease datasets, and computational prediction identified candidate regulatory interactions and compounds for further investigation. qPCR validation in clinical samples (*n* = 15 per group) showed significantly increased expression of all five hub genes in gingival tissues from patients with periodontitis and PBMCs from patients with SLE compared with their respective healthy controls.

**Conclusion:**

This study identified CCL3, LY96, FOS, MMP9, and PTGS2 as candidate genes associated with periodontitis and SLE. FOS, LY96, and PTGS2 showed consistent expression patterns across datasets, whereas CCL3 and MMP9 exhibited dataset‐dependent variation in SLE. Clinical qPCR validation showed increased expression of all five genes in both disease cohorts. These findings provide insight into the shared molecular features of periodontitis and SLE and warrant further validation in larger, independent, and clinically well‐characterized cohorts.

**Abbreviations**
ACR
: American College of Rheumatology
AUC
: area under the curve
DEGs
: differentially expressed genes
GEO
: Gene Expression Omnibus
GO
: Gene Ontology
KEGG
: Kyoto Encyclopedia of Genes and Genomes
NCBI
: National Center for Biotechnology Information
PPI
: protein–protein interaction
qPCR
: quantitative real‐time PCR
ROC
: receiver operating characteristic
SLE
: systemic lupus erythematosus
TF
: transcription factor

## 1 Introduction

Periodontitis is a chronic inflammatory disorder triggered by a microbial biofilm that forms around the teeth, leading to destruction of supporting periodontal tissues and a decline in quality of life [1]. Without timely intervention, it may result in periodontal pocket formation, bleeding on probing, progressive clinical attachment loss and alveolar bone loss, and ultimately premature tooth loss [2, 3]. In clinical practice, periodontal status is commonly evaluated using parameters such as probing depth, clinical attachment level, bleeding on probing, and radiographic evidence of alveolar bone loss, which together reflect disease severity and periodontal tissue destruction [4]. Periodontitis is one of the most prevalent chronic inflammatory diseases worldwide, affecting an estimated 20%–50% of the adult population [5, 6, 7]. A growing body of epidemiological evidence has linked periodontitis to various systemic disorders and an elevated risk of mortality [8, 9, 10]. Additionally, this condition is shaped by both genetic and environmental factors and shows a close association with several systemic inflammatory and autoimmune diseases, including systemic lupus erythematosus (SLE) [11].

SLE is a severe autoimmune disorder with an unpredictable relapsing‐remitting course, capable of inflicting widespread damage on organs such as the skin, joints, kidneys, central nervous system, and hematopoietic system [12]. Clinically, disease activity and organ involvement vary considerably among patients, requiring regular assessment and individualized management to prevent disease flares and irreversible organ damage [13]. A hallmark of SLE is the production of autoantibodies and the formation of immune complexes, which can deposit in tissues and promote inflammatory responses and organ damage [14]. As a multifactorial disease of unclear pathogenesis, SLE susceptibility is shaped by genetic and environmental factors, immune‐endocrine dysregulation, and epigenetic modifications [15, 16]. One study reported that approximately 70% of patients with SLE have periodontitis [17]. Previous studies have reported an association between periodontitis and greater disease severity or systemic inflammation in patients with SLE [18, 19]. These observations highlight the clinical relevance of periodontal health in patients with SLE and the importance of interdisciplinary awareness. Elucidating the shared molecular pathways between periodontitis and SLE may further clarify their clinical association and identify candidate molecular features underlying this association.

Although recent bioinformatics studies have investigated the molecular connections between periodontitis and SLE, the reported hub genes vary considerably depending on the datasets and analytical methods employed. In the present study, we conducted a bioinformatics screening using different GEO datasets to identify shared hub genes between these two diseases. The resulting candidate genes were subsequently evaluated in independent datasets and validated by qPCR in clinical samples, while their discriminatory performance was assessed using ROC curve analysis. Regulatory networks, immune infiltration patterns, and candidate compounds were also explored to provide further insight into the shared molecular and immune features of periodontitis and SLE.

## 2 Methods and Materials

### 2.1 Data Collection

The research design workflow is illustrated in Figure 1. The Gene Expression Omnibus (GEO) database, accessible at https://www.ncbi.nlm.nih.gov/geo/, is a public gene expression repository maintained by the National Center for Biotechnology Information (NCBI) in the United States. Using the keywords “periodontitis,” “gingiva,” and “human genome,” we retrieved two datasets, GSE16134 and GSE10334, from GEO. Similarly, the keywords “systemic lupus erythematosus,” “SLE,” and “human genome” were used to obtain the GSE61635 and GSE121239 datasets. GSE16134 and GSE61635 served as the training datasets, while GSE10334 and GSE121239 were used as independent validation datasets to evaluate the expression patterns of the identified hub genes in periodontitis and SLE. Detailed characteristics of these GEO datasets are provided in Table 1.

**Figure 1:**
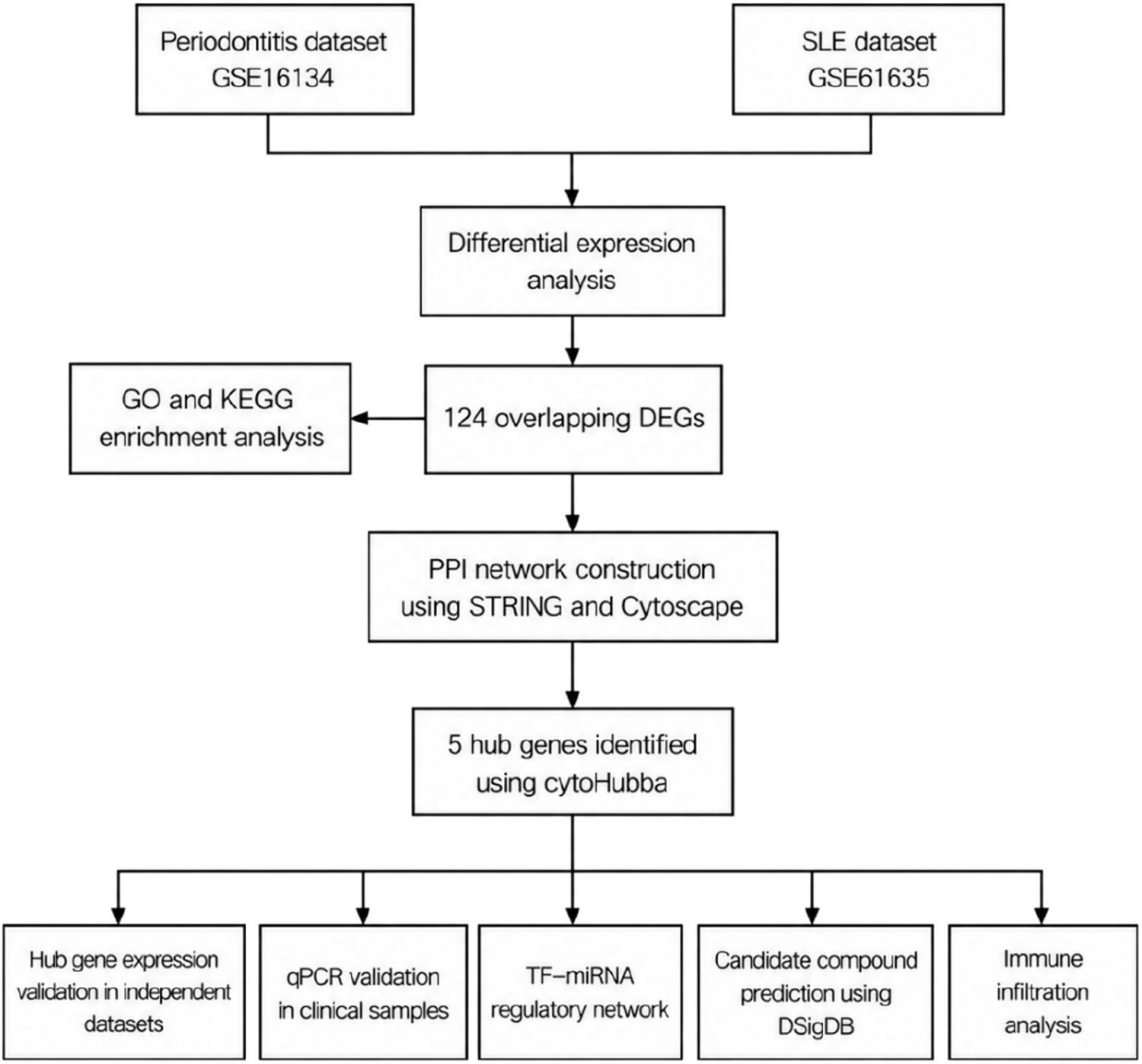
Study workflow. DEGs, differentially expressed genes; DSigDB, Drug Signatures Database; GO, Gene Ontology; KEGG, Kyoto Encyclopedia of Genes and Genomes; PPI, protein–protein interaction; qPCR, quantitative real‐time PCR; SLE, systemic lupus erythematosus; TF, transcription factor.

**Table 1:** Characteristics of the GEO datasets included in this study.

ID	Group	GSE number	Platform	Samples	Disease
1	Training dataset	GSE16134	GPL570	241 patients and 69 controls	Periodontitis
2	Validation dataset	GSE10334	GPL570	183 patients and 64 controls	Periodontitis
3	Training dataset	GSE61635	GPL570	99 patients and 30 controls	SLE
4	Validation dataset	GSE121239	GPL13158	292 patients and 20 controls	SLE

### 2.2 Differentially Expressed Gene Analysis

The “limma” R package was applied to analyze the expression profiles of GSE16134 and GSE61635 to identify differentially expressed genes (DEGs) between disease and control groups. DEGs in periodontitis and SLE were selected using the following thresholds: |log_2_(fold change)| > 0.5 and adjusted *p* < 0.05. The fold‐change threshold was selected to retain genes with moderate but statistically significant expression changes for subsequent cross‐disease screening, consistent with thresholds used in previous transcriptomic and cross‐disease bioinformatics studies [20, 21, 22], while the adjusted *p*‐value criterion was applied to control for multiple testing. Overlapping DEGs common to both conditions were determined using Venny 2.1 (https://bioinfogp.cnb.csic.es/tools/venny), an online tool for Venn diagram generation. For visualization, volcano plots were constructed using the “ggplot2” R package, and heatmaps were generated using the “ComplexHeatmap” R package.

### 2.3 GO and KEGG Functional Enrichment Analyses

To comprehensively investigate the functional relevance of the overlapping DEGs, we performed Gene Ontology (GO) and Kyoto Encyclopedia of Genes and Genomes (KEGG) enrichment analyses using the “clusterProfiler” R package. GO annotation included three categories: biological process, molecular function, and cellular component. KEGG pathway analysis was used to identify biological pathways enriched among the overlapping DEGs. Statistical significance for enrichment was defined as *p* < 0.05.

### 2.4 Protein‐Protein Interaction (PPI) Network Construction

To build a PPI network based on the overlapping DEGs, the STRING database (version 12.0, https://cn.string-db.org/) was employed. Protein–protein interactions with a minimum required interaction score of 0.4 were included in the network. The resulting network was then imported into Cytoscape software (version 3.9.1) for graphical visualization. Hub genes were identified using the cytoHubba plugin within Cytoscape, which implements four distinct algorithms: Degree, DMNC, MCC, and MNC.

### 2.5 Identification and Validation of Hub Gene Expression

The expression levels of the selected hub genes were first examined in the training datasets: GSE16134 (periodontitis) and GSE61635 (SLE). Differences in expression between patient and healthy control groups were assessed. Subsequently, the expression patterns of the hub genes were evaluated in the independent validation datasets GSE10334 (periodontitis) and GSE121239 (SLE). The expression patterns were additionally visualized using box plots. A *p* < 0.05 was considered statistically significant for all comparisons between patients and controls.

To evaluate the discriminatory performance of the identified hub genes in periodontitis and SLE, receiver operating characteristic (ROC) curve analysis was performed. The area under the curve (AUC) was calculated for each hub gene in both the training and validation datasets. ROC analysis was conducted using the “pROC” R package, and the resulting curves were visualized using the “ggplot2” R package.

### 2.6 Transcription Factor and miRNA Coregulatory Network

The NetworkAnalyst 3.0 platform (https://www.networkanalyst.ca) was used to predict potential regulatory interactions of the hub genes with transcription factors (TFs) and miRNAs. TF–hub gene interactions were obtained from the TRRUST database, while potential miRNA–hub gene interactions were obtained from the miRTarBase database. The resulting TF–hub gene and miRNA–hub gene regulatory networks were subsequently constructed and visualized using Cytoscape.

### 2.7 Analysis of Immune Cell Infiltration

Gene expression profiles from the periodontitis and SLE training datasets were analyzed using the CIBERSORT algorithm to estimate the relative proportions of 22 immune cell subsets. Violin plots were used to compare the estimated proportions of immune cell subsets between disease and healthy control groups in each training dataset. Pearson correlation analysis was performed to assess the relationships between hub gene expression and the estimated proportions of immune cell subsets. The “corrplot” R package was used to generate heatmaps illustrating these correlations. A *p* < 0.05 was considered statistically significant.

### 2.8 Prediction of Candidate Compounds

The Drug Signatures Database (DSigDB, http://dsigdb.tanlab.org/DSigDBv1.0/) was used to predict candidate compounds potentially associated with the identified hub genes. Information on the predicted candidate compounds, including PubChem CID and molecular formula, was retrieved from the PubChem database (https://pubchem.ncbi.nlm.nih.gov/).

### 2.9 Specimen Collection

All participants were enrolled at the First Affiliated Hospital of Nanchang University between November 2024 and August 2026. The demographic and clinical characteristics of the enrolled participants, including age, sex, and relevant disease‐specific clinical parameters, are summarized in Table S1.

For the SLE cohort, peripheral blood samples were collected from 30 participants, including 15 patients with SLE and 15 healthy controls. SLE diagnosis was based on the 1997 American College of Rheumatology (ACR) criteria. All patients with SLE were treatment‐naïve at the time of blood collection and had not received glucocorticoids, immunosuppressants, or other immunomodulatory therapies before sample collection. Information on disease duration and disease activity is summarized in Table S1. The healthy controls had no history of autoimmune or inflammatory diseases.

For the periodontitis cohort, gingival tissue samples were obtained from 30 participants, including 15 patients with periodontitis and 15 healthy controls. Periodontal health was defined as a gingival index of 0 (no clinical inflammation), probing depth ≤ 3 mm, no clinical attachment loss, and no radiographic bone loss. Periodontitis was defined as a gingival index > 1, probing depth ≥ 5 mm, clinical attachment loss ≥ 3 mm, and radiographic bone loss. Inflamed gingival tissues were collected from patients with periodontitis during periodontal flap surgery or tooth extraction performed for periodontal reasons, whereas healthy gingival tissues were obtained from healthy controls during extraction of impacted wisdom teeth. All gingival tissue samples measured approximately 2 mm^3^ and were immediately placed in RNA stabilization reagent.

This study was conducted in accordance with the Declaration of Helsinki and was approved by the Medical Ethics Committee of the First Affiliated Hospital of Nanchang University in November 2024 (Approval No. IITS2024753), prior to participant enrollment and sample collection. All participants were fully informed about the study and provided written informed consent.

### 2.10 RNA Extraction

Peripheral blood samples (10 mL each) were collected into EDTA‐containing tubes from 15 patients with SLE and 15 healthy controls. Peripheral blood mononuclear cells (PBMCs) were separated using Ficoll density gradient centrifugation within 4 h of blood collection. Total RNA was isolated from both PBMCs and gingival tissues using the TRIzol reagent (Thermo Fisher Scientific, Waltham, MA, USA), following the manufacturer's instructions. RNA concentration and purity were assessed using a NanoDrop ND‐1000 spectrophotometer (Thermo Fisher Scientific, Wilmington, DE, USA). Purified RNA was subsequently stored at −80°C until further analysis.

### 2.11 Quantitative Real‐Time PCR

Total RNA was reverse‐transcribed into complementary DNA (cDNA) using the FastKing RT Kit (KR116, TIANGEN, Beijing, China). Quantitative real‐time PCR (qPCR) was performed on a Bio‐Rad CFX96 Touch thermal cycler (Hercules, CA, USA) using FastReal qPCR PreMix containing SYBR Green (FP217, TIANGEN, Beijing, China). The thermal cycling conditions consisted of an initial denaturation at 95°C for 3 min, followed by 40 cycles of 95°C for 15 s and 60°C for 30 s. GAPDH was used as the internal reference gene, and relative mRNA expression levels were calculated using the 2^^−ΔΔCt^ method. Primer sequences used in this study are provided in Table 2. All qPCR reactions were performed in triplicate.

**Table 2:** Primer sequences used for qPCR.

Gene	Forward primer (5′–3′)	Reverse primer (5′–3′)
CCL3	ACCAGTTCTCTGCATCACTTGCT	TGCTCGTCTCAAAGTAGTCAGC
LY96	CCGAGGATCT GATGACGATTAC	TGAATTAGGTTGGTGTAGGATGAC
FOS	CGTGCCAGACATGGACCTAT	CGGGGTAGGTGAAGACGAAG
MMP9	TTGACAGCGACAAGAAGTGG	CCTCAGTGAAGCGGTACATAG
PTGS2	GGAAGCACTCTATGGTGACATC	CCAGTAGGCAGGAGAACATATAAC
GAPDH	TGCACCACCAACTGCTTAGC	GGCATGGACTGTGGTCATGAG

### 2.12 Statistical Analysis

All statistical analyses were performed using R (version 4.2.2) and GraphPad Prism (version 9.5.1). Continuous variables were assessed for normality. Normally distributed continuous variables were compared between two groups using the independent two‐sample Student's *t*‐test, whereas non‐normally distributed continuous variables were analyzed using the Mann–Whitney *U* test. Categorical variables were compared using the chi‐square test or Fisher's exact test, as appropriate. Pearson correlation analysis was used to assess correlations between continuous variables. All statistical tests were two‐tailed, and *p* < 0.05 was considered statistically significant.

## 3 Results

### 3.1 Identification of Overlapping DEGs Between Periodontitis and SLE

Using the “limma” R package, we identified 1151 DEGs in the GSE16134 dataset, including 689 upregulated and 462 downregulated genes. The corresponding volcano plot is shown in Figure 2A, and the heatmap in Figure 2B displays the top 10 upregulated and top 10 downregulated genes. In the GSE61635 dataset, 3617 DEGs were identified, including 1999 upregulated and 1618 downregulated genes. The corresponding volcano plot and heatmap are shown in Figure 2C,D, respectively. Venn analysis identified 124 DEGs shared between periodontitis and SLE, including 73 commonly upregulated and 51 commonly downregulated genes (Figure 2E,F).

**Figure 2:**
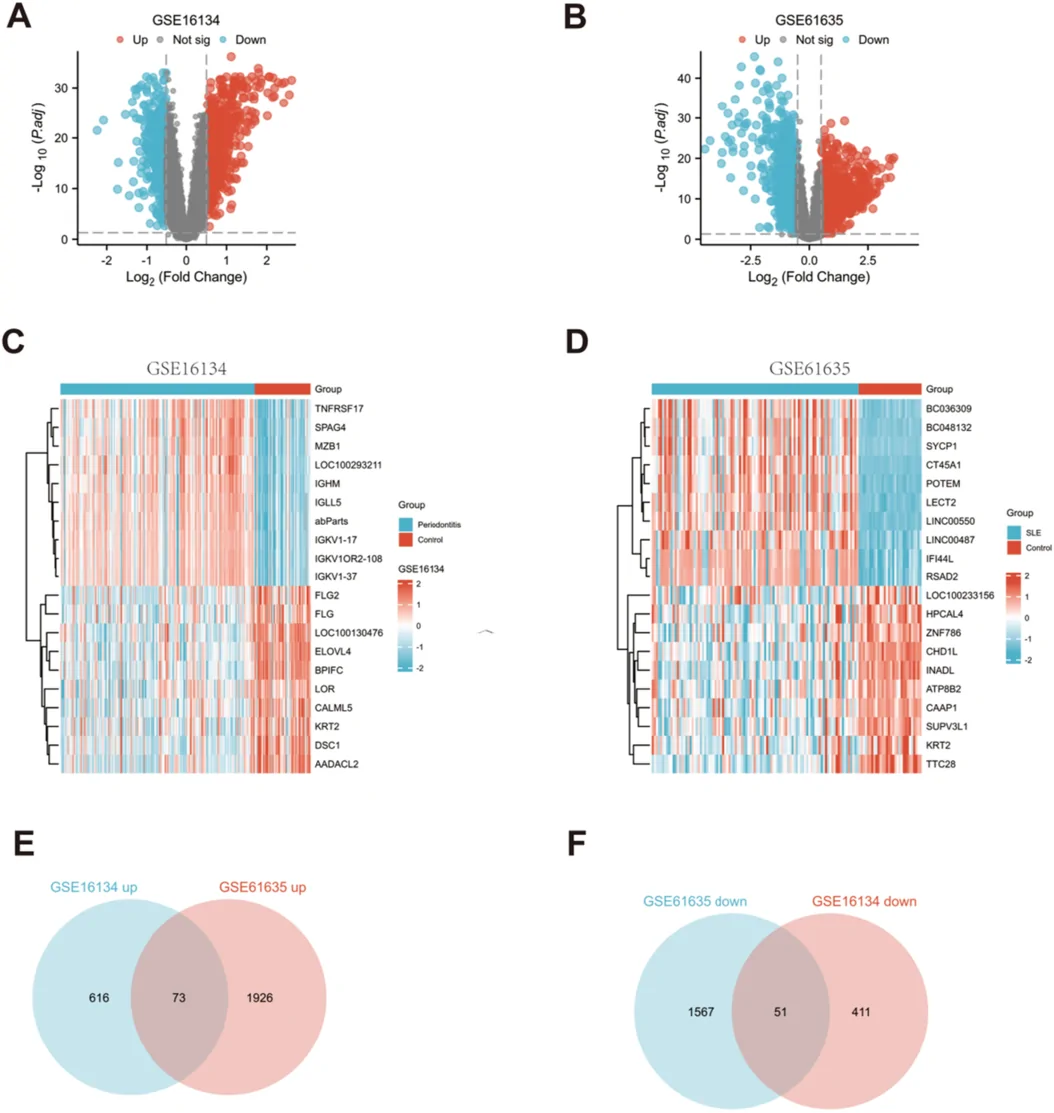
Identification of differentially expressed genes (DEGs) in the periodontitis and SLE datasets. (A, B) Volcano plot and heatmap showing DEGs in GSE16134. (C, D) Volcano plot and heatmap showing DEGs in GSE61635. (E, F) Venn diagrams showing the overlapping DEGs between the two datasets, including 73 commonly upregulated and 51 commonly downregulated genes.

### 3.2 Functional Enrichment Analysis of Overlapping DEGs

We performed GO and KEGG enrichment analyses of the overlapping DEGs and listed the top 10 GO terms and KEGG pathways (Figure 3A,B). GO enrichment analysis indicated that these DEGs were predominantly associated with regulation of the inflammatory response, positive regulation of the response to external stimuli, activation of the immune response, leukocyte chemotaxis, leukocyte migration, regulation of leukocyte chemotaxis, positive regulation of lymphocyte chemotaxis, the chemokine‐mediated signaling pathway, regulation of lymphocyte chemotaxis, and positive regulation of leukocyte chemotaxis. KEGG enrichment analysis revealed significant enrichment in multiple pathways, including lipid and atherosclerosis, the Toll‐like receptor signaling pathway, Chagas disease, pertussis, alcoholic liver disease, the TNF signaling pathway, the NF‐κB signaling pathway, the IL‐17 signaling pathway, rheumatoid arthritis, and pantothenate and CoA biosynthesis. Collectively, these enrichment results suggest that the overlapping DEGs are predominantly associated with inflammatory and immune‐related biological processes and signaling pathways.

**Figure 3:**
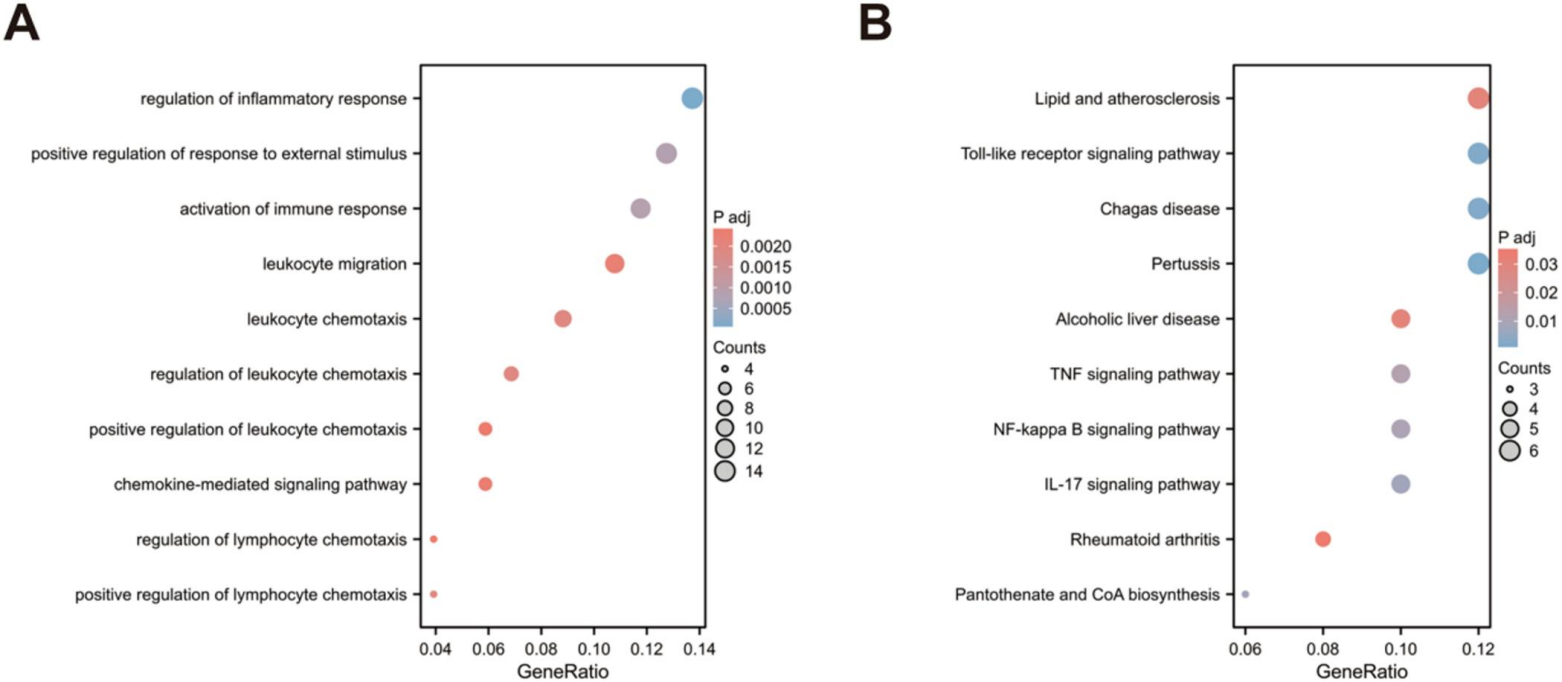
GO and KEGG enrichment analyses of the overlapping DEGs. (A) GO enrichment analysis of the overlapping DEGs. (B) KEGG pathway enrichment analysis of the overlapping DEGs.

### 3.3 PPI Network Construction and Hub Gene Identification

A PPI network of the overlapping DEGs was built using the STRING database and then imported into Cytoscape for visualization. Analysis of the 124 overlapping genes using a minimum required interaction score of 0.4 yielded a network comprising 119 nodes and 135 edges (Figure 4A). Four algorithms (Degree, DMNC, MCC, and MNC) implemented in the cytoHubba plugin within Cytoscape were applied to screen for hub genes, leading to the identification of five candidate hub genes: CCL3, LY96, FOS, MMP9, and PTGS2 (Figure 4B–F). These hub genes were considered potentially involved in the molecular link between periodontitis and SLE.

**Figure 4:**
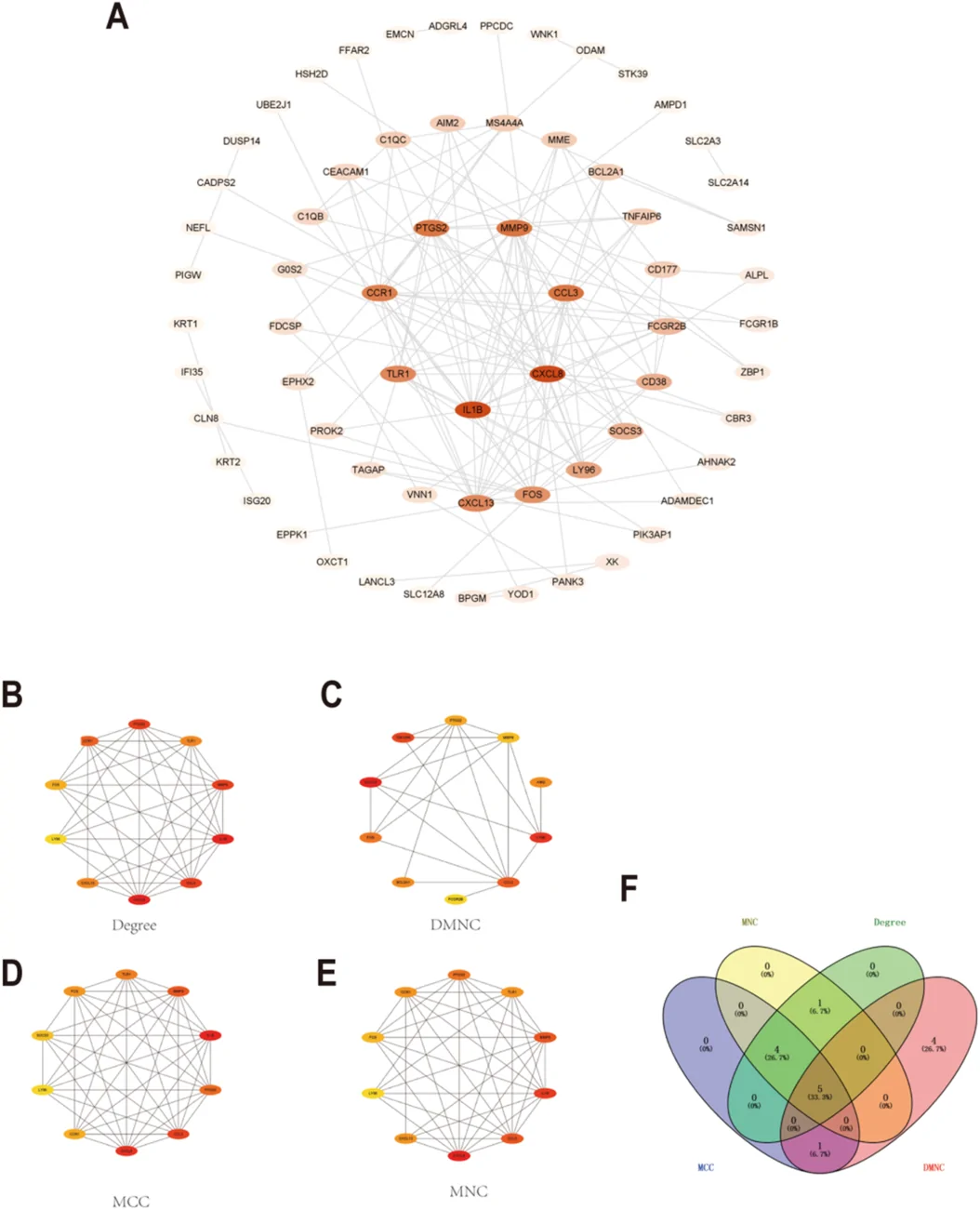
Identification of hub genes. (A) Protein–protein interaction (PPI) network of the overlapping DEGs. (B–E) Top 10 candidate genes ranked by the Degree, DMNC, MCC, and MNC algorithms, respectively. (F) Venn diagram showing five overlapping hub genes (CCL3, LY96, FOS, MMP9, and PTGS2) identified by the four algorithms.

### 3.4 Validation of Hub Genes

The expression levels of the five hub genes (CCL3, LY96, FOS, MMP9, and PTGS2) differed significantly between disease and control samples in both the periodontitis and SLE training datasets (Figure 5A,B). Their expression patterns were further evaluated in the independent validation datasets GSE10334 and GSE121239. In the periodontitis validation dataset GSE10334, all five hub genes showed differential expression patterns consistent with those observed in the training dataset (Figure 5C). In the SLE validation dataset GSE121239, FOS, LY96, and PTGS2 showed consistent differential expression, whereas CCL3 and MMP9 did not differ significantly between SLE and control samples (Figure 5D). These findings indicate that three of the five hub genes were consistently validated across the SLE datasets, whereas CCL3 and MMP9 exhibited dataset‐dependent variation.

**Figure 5:**
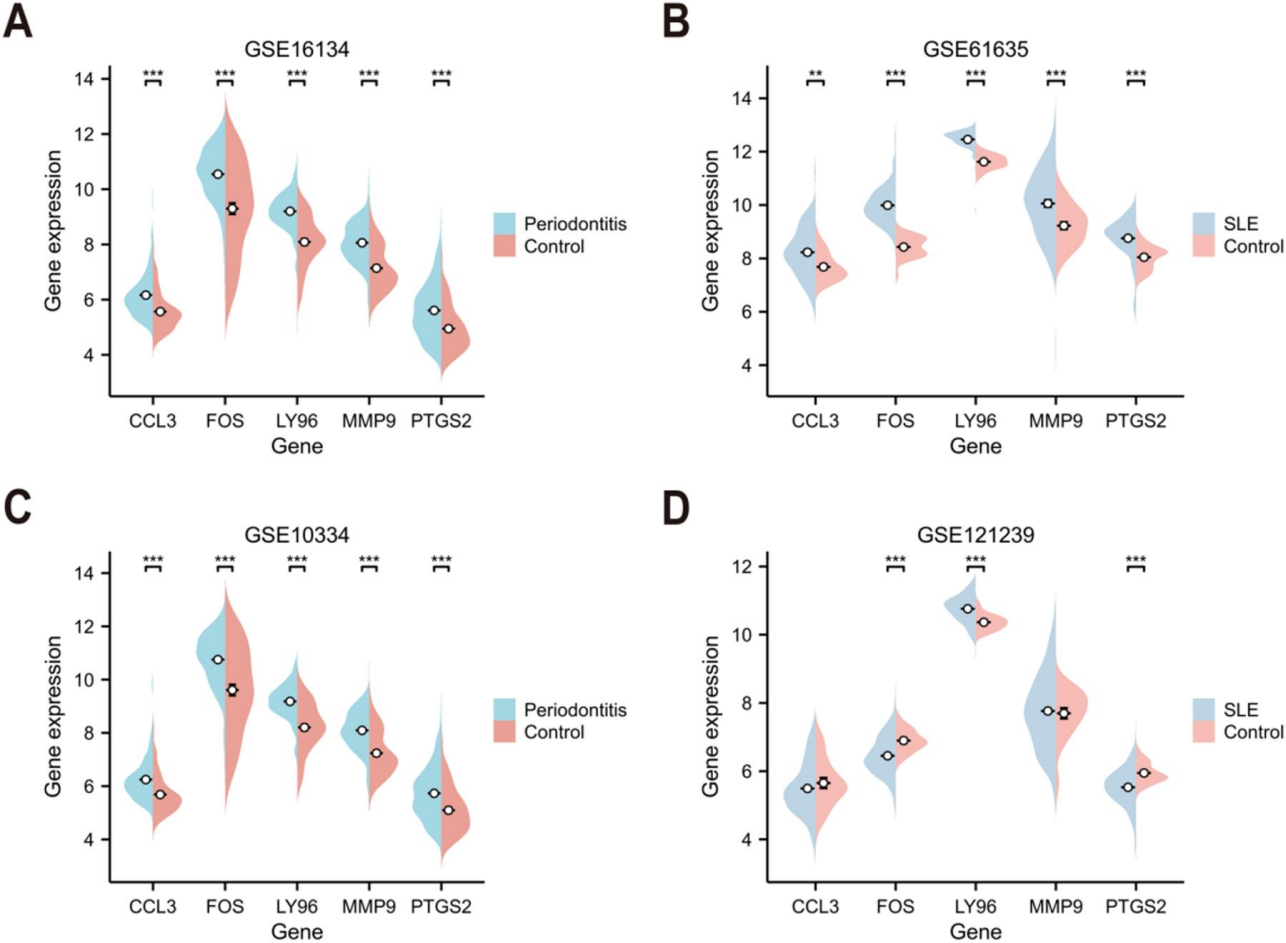
Expression validation of the five hub genes in the training and independent validation datasets. (A, B) Expression levels of CCL3, LY96, FOS, MMP9, and PTGS2 in the periodontitis training dataset GSE16134 and SLE training dataset GSE61635, respectively. (C, D) Expression levels of the five hub genes in the independent validation datasets GSE10334 (periodontitis) and GSE121239 (SLE), respectively. SLE, systemic lupus erythematosus.

### 3.5 ROC Analysis of the Hub Genes

Receiver operating characteristic (ROC) curve analysis was performed to evaluate the discriminatory performance of the hub genes across different datasets. In the periodontitis training dataset GSE16134, the area under the curve (AUC) values for CCL3, FOS, LY96, MMP9, and PTGS2 were 0.734, 0.724, 0.844, 0.796, and 0.694, respectively (Figure 6A). Similarly, in the SLE training dataset GSE61635, the corresponding AUC values were 0.696, 0.993, 0.969, 0.721, and 0.840, respectively (Figure 6B). These results indicate varying degrees of discriminatory performance among the five hub genes in the training datasets.

**Figure 6:**
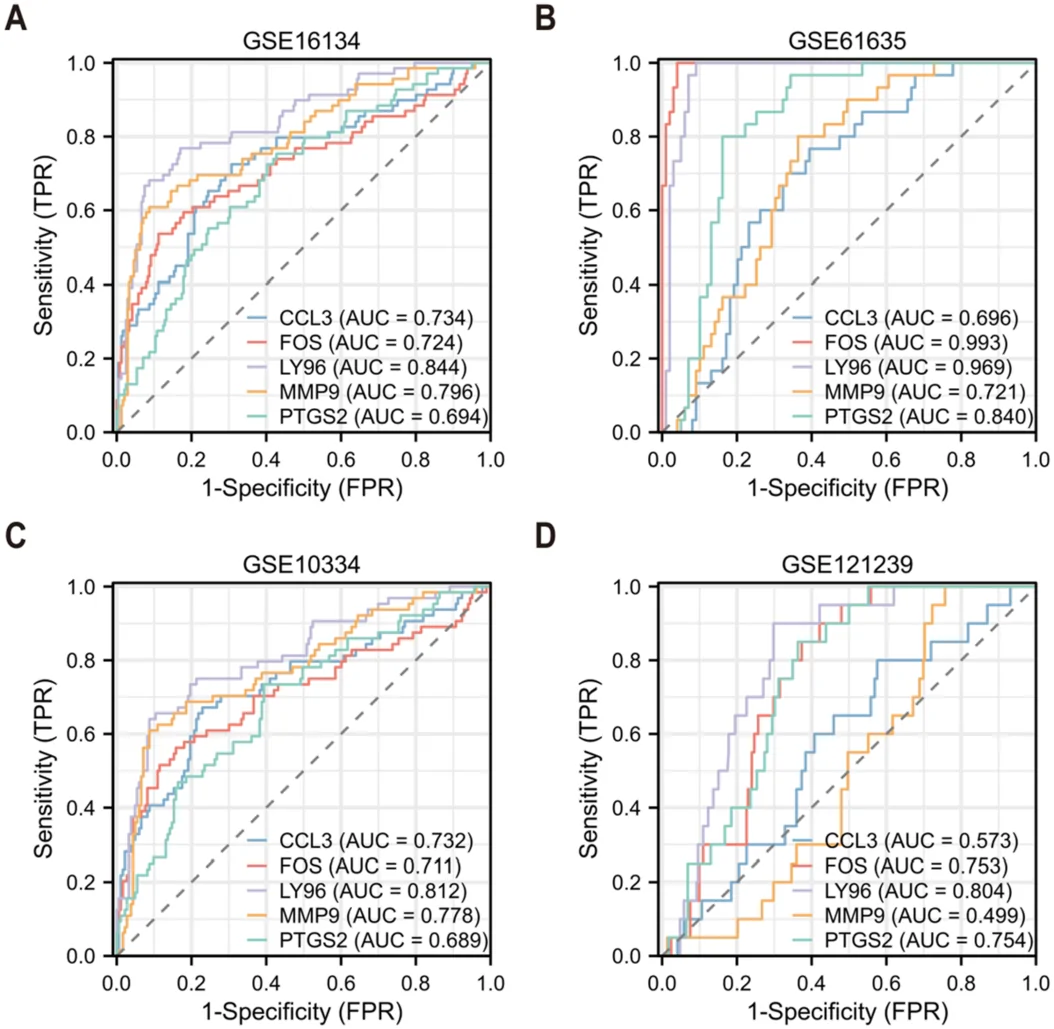
Discriminatory performance of the five hub genes in the training and validation datasets. Receiver operating characteristic (ROC) curve analyses of CCL3, LY96, FOS, MMP9, and PTGS2 in (A) GSE16134 (periodontitis training dataset), (B) GSE61635 (SLE training dataset), (C) GSE10334 (periodontitis validation dataset), and (D) GSE121239 (SLE validation dataset). AUC, area under the curve; ROC, receiver operating characteristic; SLE, systemic lupus erythematosus.

In the periodontitis validation dataset GSE10334, the AUC values for CCL3, FOS, LY96, MMP9, and PTGS2 were 0.732, 0.711, 0.812, 0.778, and 0.689, respectively (Figure 6C). In the SLE validation dataset GSE121239, the corresponding AUC values were 0.573, 0.753, 0.804, 0.499, and 0.754, respectively (Figure 6D). FOS, LY96, and PTGS2 showed relatively higher discriminatory performance in the SLE validation dataset, whereas CCL3 and MMP9 exhibited limited discriminatory ability. Overall, the ROC analyses demonstrated variable discriminatory performance of the identified hub genes across the analyzed datasets, and their potential clinical utility requires further validation in larger, independent cohorts.

### 3.6 Clinical Validation of Hub Genes by Qpcr

To further validate the expression of the identified hub genes in clinical samples, qPCR analysis was performed with 15 participants per group. The relative mRNA expression levels of CCL3, LY96, FOS, MMP9, and PTGS2 were significantly elevated in gingival tissues from patients with periodontitis compared with healthy controls (Figure 7A–E). Similarly, all five hub genes showed significantly increased mRNA expression in PBMCs from patients with SLE compared with healthy controls (Figure 7F–J).

**Figure 7:**
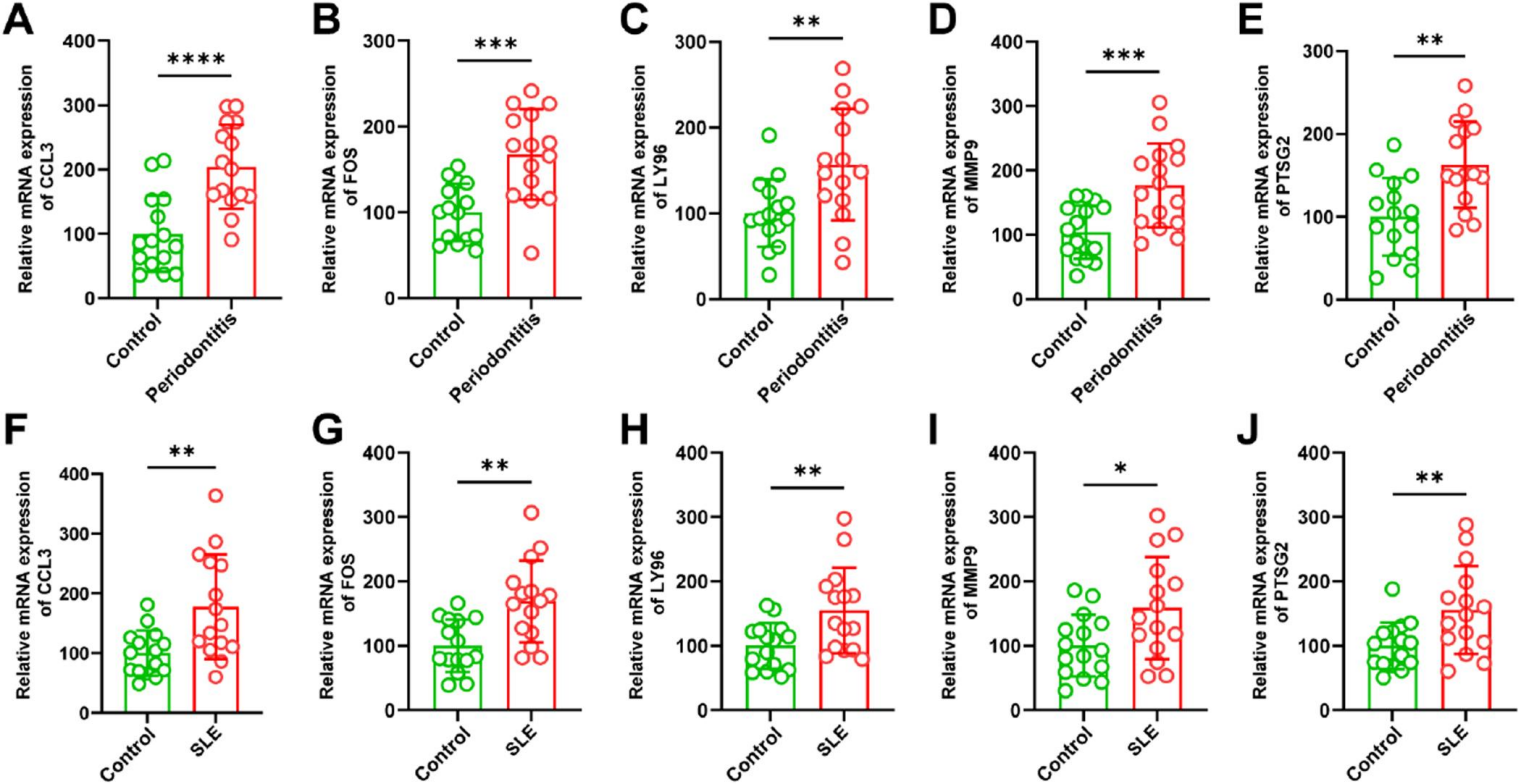
Clinical validation of the five hub genes by quantitative real‐time PCR (qPCR). (A–E) Relative mRNA expression levels of CCL3, LY96, FOS, MMP9, and PTGS2 in gingival tissues from patients with periodontitis and healthy controls (*n* = 15 per group). (F–J) Relative mRNA expression levels of CCL3, LY96, FOS, MMP9, and PTGS2 in PBMCs from patients with SLE and healthy controls (*n* = 15 per group). Data are presented as mean ± SD. *p < 0.05, **p < 0.01, ***p < 0.001, and **p < 0.0001. PBMCs, peripheral blood mononuclear cells; SLE, systemic lupus erythematosus.

### 3.7 Regulatory Network of Transcription Factors and miRNAs

Database‐based prediction identified 90 TFs and 102 miRNAs potentially associated with the five hub genes (Figure 8). The resulting regulatory network comprised 197 nodes and 228 edges. Within this network, the connectivity degrees for CCL3, LY96, FOS, MMP9, and PTGS2 were 2, 95, 63, 8, and 62, respectively. Overall, the predicted network revealed extensive potential regulatory associations among TFs, hub genes, and miRNAs.

**Figure 8:**
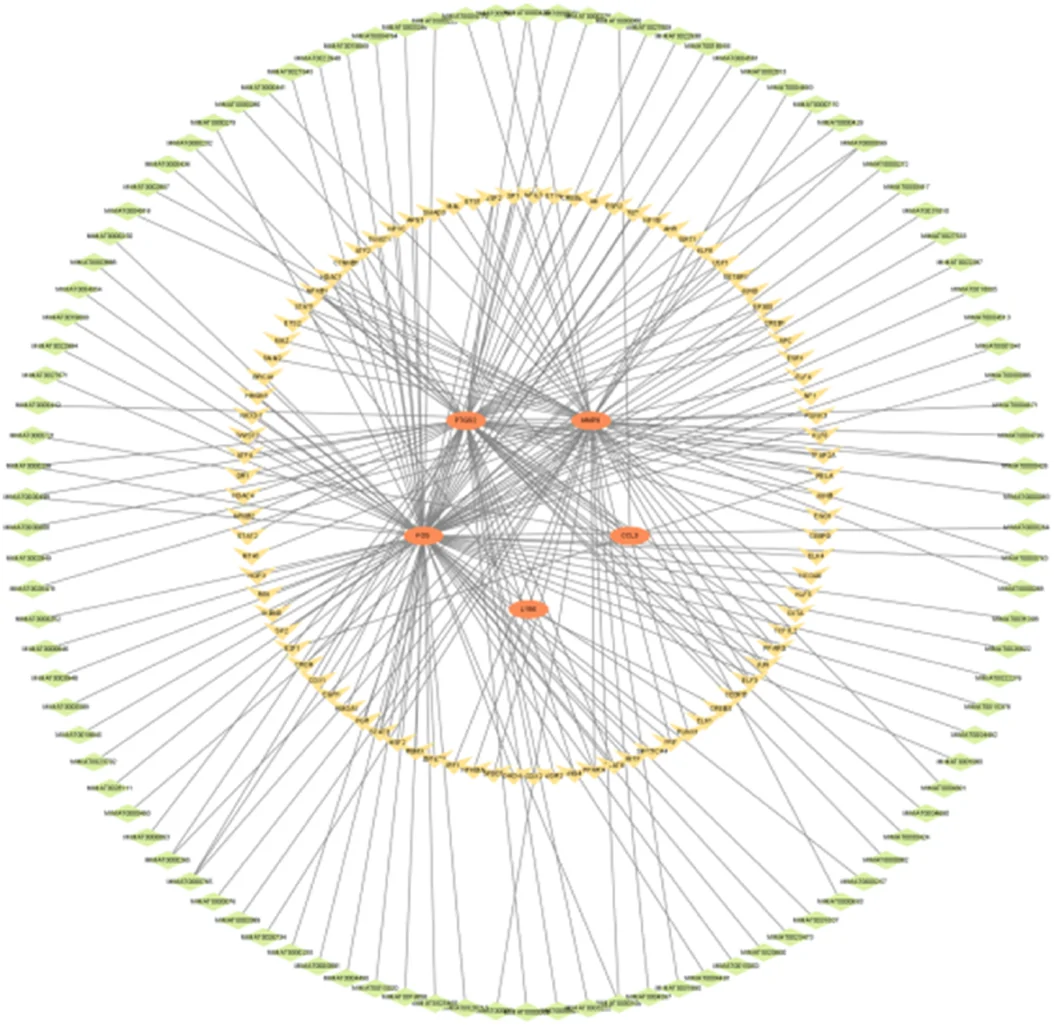
Regulatory network of transcription factors (TFs) and miRNAs. In the network, orange nodes represent hub genes, yellow nodes represent TFs, and green nodes represent miRNAs. miRNA, microRNA; TF, transcription factor.

### 3.8 Immune Infiltration Analysis

To characterize immune cell infiltration in periodontitis and SLE, the CIBERSORT algorithm was applied to the training datasets to estimate the relative proportions of 22 immune cell subsets. In the GSE16134 dataset, significant differences between periodontitis and control samples were observed in multiple immune cell subsets, including naive B cells, memory B cells, plasma cells, activated memory CD4^+^ T cells, follicular helper T cells, γδ T cells, activated natural killer (NK) cells, M0 macrophages, M1 macrophages, resting dendritic cells, activated dendritic cells, resting mast cells, and neutrophils (Figure 9A). In the GSE61635 dataset, significant differences between SLE and control samples were observed in naive B cells, plasma cells, CD8^+^ T cells, resting memory CD4^+^ T cells, activated memory CD4^+^ T cells, monocytes, activated dendritic cells, and neutrophils (Figure 9B). Among these, differences in the estimated proportions of naive B cells, plasma cells, activated memory CD4^+^ T cells, activated dendritic cells, and neutrophils were observed in both disease datasets relative to their respective control groups.

**Figure 9:**
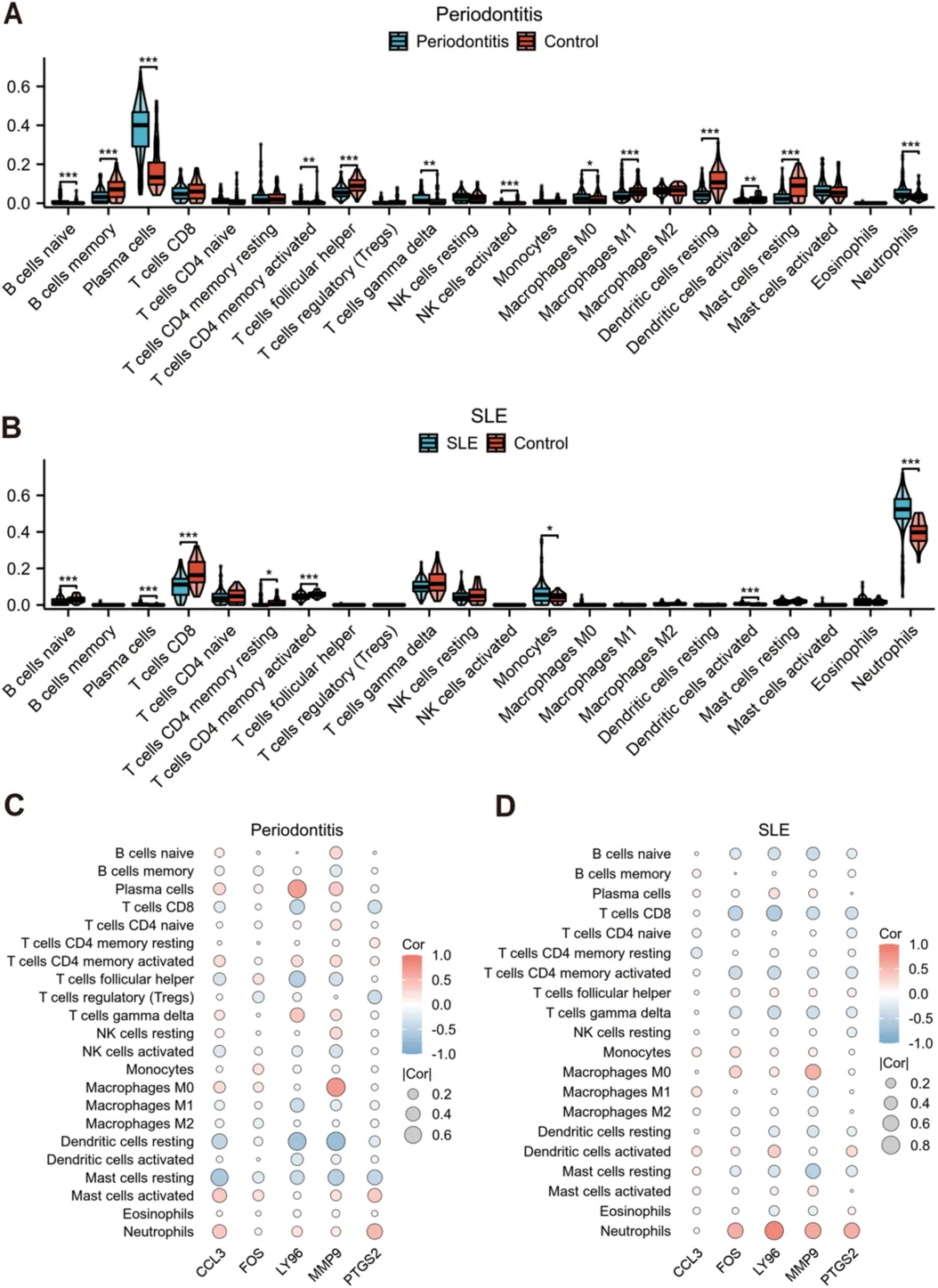
Immune cell infiltration and correlations with hub gene expression in periodontitis and SLE. (A, B) Violin plots showing the relative proportions of 22 immune cell subsets in the periodontitis (GSE16134) and SLE (GSE61635) datasets, respectively. (C, D) Heatmaps showing the correlations between hub gene expression and infiltrating immune cell subsets in periodontitis and SLE, respectively. The color gradient represents the direction and strength of the correlation, ranging from positive (red) to negative (blue). SLE, systemic lupus erythematosus.

### 3.9 Correlations Between Immune Cells and Hub Genes

To further explore the relationships between immune cell infiltration and the five hub genes in periodontitis and SLE, correlation analyses were performed (Figure 9C,D). In the periodontitis dataset, the five hub genes exhibited distinct patterns of association with multiple immune cell subsets (Figure 9C). Similarly, heterogeneous correlation patterns between hub gene expression and immune cell infiltration were observed in the SLE dataset (Figure 9D). The direction and magnitude of these correlations varied across individual hub genes and immune cell subsets. Overall, these findings indicate associations between hub gene expression and distinct patterns of estimated immune cell infiltration in periodontitis and SLE.

### 3.10 Prediction of Candidate Compounds

Candidate compounds were computationally predicted using the DSigDB database. The top 10 predicted compounds were dinoprostone, pyrrolidine dithiocarbamate, capsaicin, phencyclidine, flurbiprofen, propranolol, simvastatin, bisindolylmaleimide, DL‐mevalonic acid, and beclomethasone (Table 3).

**Table 3:** Top 10 predicted candidate compounds.

Candidate compounds	PubChem CID	Molecular formula	Combined score	*p* value	Genes
Dinoprostone	5280360	C20H32O5	12071.996	7.71386E‐09	CCL3; FOS; PTGS2; MMP9
Pyrrolidine dithiocarbamate	65351	C5H9NS2	9099.767	1.91114E‐08	CCL3; FOS; PTGS2; MMP9
Capsaicin	1548943	C18H27NO3	6557.263	5.42182E‐08	CCL3; FOS; PTGS2; MMP9
Phencyclidine	6468	C17H25N	5827.801	7.86854E‐08	CCL3; FOS; PTGS2; MMP9
Flurbiprofen	3394	C15H13FO2	11443.723	1.06093E‐07	FOS; PTGS2; MMP9
Propranolol	4946	C16H21NO2	10018.350	1.46487E‐07	CCL3; PTGS2; MMP9
Simvastatin	54454	C25H38O5	3983.143	2.5857E‐07	CCL3; FOS; PTGS2; MMP9
Bisindolylmaleimide	2396	C25H24N4O2	7491.173	2.96506E‐07	FOS; PTGS2; MMP9
DL‐Mevalonic acid	449	C6H12O4	7202.927	3.26092E‐07	CCL3; PTGS2; MMP9
Beclomethasone	20469	C22H29ClO5	6934.075	3.57581E‐07	CCL3; FOS; MMP9

## 4 Discussion

Periodontitis and SLE are inflammatory diseases with a well‐recognized clinical association [19, 23]. Although previous bioinformatics studies have explored their molecular links using public datasets [18, 24], experimental validation of the proposed shared molecular signatures remains limited. In this study, we independently screened for shared hub genes using different GEO datasets, further evaluated their expression patterns in independent datasets, and validated their expression by qPCR in clinical samples. Our findings are consistent with several previously reported associations, including those involving LY96 and MMP9 [18, 24], while also identifying a combination of five hub genes (CCL3, LY96, FOS, MMP9, and PTGS2) that, to our knowledge, has not been collectively reported previously. These findings provide additional evidence for shared molecular features between periodontitis and SLE and identify candidate genes that warrant further investigation for their potential clinical relevance.

CCL3, also known as macrophage inflammatory protein‐1α (MIP‐1α), is a key mediator of inflammatory responses [25]. As a member of the CC chemokine family, CCL3 is highly expressed in monocytes, macrophages, neutrophils, and other immune cells during inflammation [26]. CCL3 has been associated with osteolytic bone disease and increased bone resorption. Proinflammatory stimuli such as lipopolysaccharide (LPS) induce CCL3 expression, which in turn impairs osteoblast differentiation by modulating B cells, thereby suppressing bone formation [27]. In patients with SLE, elevated CCL3 expression has been reported and may contribute to the recruitment of inflammatory cells [28]. Moreover, CCL3 expression increases with periodontal disease severity, paralleling an increased lymphocyte proportion in diseased periodontal tissues [29]. In the present study, CCL3 showed increased expression in clinical samples from both periodontitis and SLE; however, its differential expression was not replicated in the independent SLE dataset GSE121239, suggesting that its association with SLE may be influenced by dataset‐ or cohort‐specific factors.

Matrix metalloproteinase 9 (MMP9), one of the largest members of the gelatinase subfamily within the MMP family, is primarily secreted by neutrophils and, to a lesser extent, by activated macrophages, T and B lymphocytes, endothelial cells, and other cell types [30]. This enzyme has been implicated in various pathological processes, including extracellular matrix degradation, tissue remodeling, and inflammatory responses [31]. Elevated serum levels of MMP9 have been reported in SLE patients compared with healthy controls [32, 33]. MMP9 is thought to contribute to vascular wall injury and an increased risk of ischemia‐reperfusion injury in SLE patients, as well as to neuropathy [34]. In periodontal tissues, MMP9 participates in the degradation of various proteins, such as collagen types IV, V, and XI, and is abundantly expressed during active stages of periodontitis [35, 36]. In the present study, MMP9 was upregulated in clinical samples from both diseases but was not consistently validated in the independent SLE dataset GSE121239, suggesting dataset‐dependent variation in SLE.

PTGS2, also known as cyclooxygenase‐2 (COX‐2), is an enzyme induced by inflammatory stimuli across various cell types, including endothelial, epithelial, and immune cells [37]. It plays an important role in inflammatory responses through the production of pro‐inflammatory prostaglandins [38, 39]. In murine models of SLE, spontaneous overexpression of COX‐2 has been reported, and administration of COX‐2 inhibitors reduces autoantibody production and suppresses T‐cell responses to major lupus autoantigens [40]. Additionally, a previous study demonstrated that COX‐2 expression in periodontal tissues correlates with the degree of inflammation in patients with chronic periodontitis [41]. In the present study, PTGS2 was consistently validated across datasets and upregulated in clinical samples from both diseases, suggesting its potential involvement in their shared inflammatory features.

The FOS gene belongs to the immediate early gene family and is transiently activated by extracellular signals such as growth factors, hormones, and cytokines [42]. The protein product of FOS can dimerize with JUN family members to form the transcription factor complex activator protein‐1 (AP‐1), which binds to DNA and participates in signal transduction, cell proliferation, differentiation, and apoptosis [43]. Several studies have implicated AP‐1‐mediated signaling in the dysregulation of IL‐2 expression and T‐cell responses in SLE [44, 45]. In addition, AP‐1 expression is markedly upregulated in antibody‐secreting cells (ASCs) from SLE patients [46]. FOS has also been identified as a differentially expressed gene in inflamed periodontal tissues compared with healthy controls [47]. FOS was consistently validated across datasets and clinical samples, supporting its potential relevance to both diseases.

Lymphocyte antigen 96 (LY96), also known as MD‐2, is a key player in innate immunity and has been implicated in diverse immune responses [48]. MD‐2 associates with Toll‐like receptor 4 (TLR4) to facilitate the recognition of LPS [49]. This complex subsequently recruits the intracellular adaptor myeloid differentiation factor, leading to activation of the NF‐κB pathway and subsequent release of proinflammatory cytokines [50]. Previous bioinformatics studies have identified LY96 as a potential biomarker associated with SLE [51]. Additionally, LY96 has been linked to *Porphyromonas gingivalis*, and its expression is significantly elevated in gingival tissues from patients with periodontitis [52]. LY96 was consistently validated across datasets and clinical samples in the present study, supporting its potential relevance to both diseases.

The inconsistent validation of CCL3 and MMP9 in GSE121239 may reflect heterogeneity across the SLE datasets, including differences in study populations, disease activity or severity, treatment status, and experimental platforms. As detailed clinical information was not uniformly available across the public datasets, the contribution of these factors could not be determined. Therefore, the associations of CCL3 and MMP9 with SLE should be interpreted cautiously and further validated in independent, clinically well‐characterized cohorts.

Enrichment analysis revealed potential molecular links between periodontitis and SLE, with significant enrichment in the Toll‐like receptor (TLR), IL‐17, and NF‐κB signaling pathways. Genome‐wide association studies, experimental mouse models, and clinical sample analyses have implicated TLRs in SLE pathogenesis [53]. In SLE patients, cytokine release accelerates B‐cell differentiation into plasma cells, which in turn enhances TLR signaling within B cells [54]. TLR signaling has also been implicated in periodontal inflammation and alveolar bone loss [55]. Furthermore, plasma and target organ levels of IL‐17 are significantly elevated in SLE patients compared with healthy controls, and its expression correlates positively with disease severity [56]. In animal studies, reducing IL‐17 levels effectively ameliorates SLE‐associated pathological changes, an effect linked to IL‐17 interactions with various T and B cells [57]. IL‐17 can also activate the NF‐κB signaling pathway and initiate bone resorption in periodontitis [58]. The NF‐κB pathway regulates proliferation and osteogenic differentiation of human periodontal ligament stem cells while promoting apoptosis under inflammatory conditions [59]. Collectively, these findings support the potential involvement of TLR, IL‐17, and NF‐κB signaling in the shared inflammatory features of periodontitis and SLE.

Immune cell infiltration analysis revealed altered immune profiles in both periodontitis and SLE, with differences in the estimated proportions of several immune cell subsets, including naive B cells, plasma cells, activated memory CD4^+^ T cells, activated dendritic cells, and neutrophils, observed in both disease datasets. Both innate and adaptive immune responses are known to contribute to the pathogenesis of periodontitis and SLE [60]. Neutrophils, the most abundant leukocytes, play important roles in inflammation, and their extracellular traps may promote SLE autoimmunity [61]. In periodontitis, neutrophils are first responders against pathogens, using antimicrobial peptides, enzymes, and reactive oxygen species [62]. Periodontal tissues also exhibit alterations in plasma cells, naive B cells, neutrophils, and activated memory CD4^+^ T cells [63]. In SLE, dysregulated B‐ and T‐cell responses contribute to systemic immune dysfunction and autoantibody production [64, 65, 66]. Activated dendritic cells can recruit T and NK cells to periodontal infection sites, and their interactions with CD4^+^ T cells further support the involvement of adaptive immunity in periodontitis [67, 68]. Correlation analysis further revealed distinct associations between hub gene expression and multiple immune cell subsets, suggesting potential relationships between the identified hub genes and immune dysregulation in both diseases.

An important consideration is that the transcriptomic datasets used for periodontitis and SLE were derived from different biological sources, primarily gingival tissues and peripheral blood/PBMCs, respectively. Gingival tissue comprises multiple resident and infiltrating cell populations, whereas peripheral blood/PBMC samples predominantly represent circulating immune cells. These differences in tissue composition and cellular context may influence gene expression profiles and, consequently, the identification of overlapping DEGs [69]. They may also affect CIBERSORT‐based immune infiltration estimates [70, 71], which should therefore be interpreted as tissue‐specific relative immune patterns rather than directly comparable immune cell abundances between periodontitis and SLE. Nevertheless, the molecular and immune signatures identified across these distinct tissue contexts may provide insight into potential common features of the two diseases and warrant further validation using more comparable biological sources and independent cohorts.

This study further explored potential TF–miRNA regulatory interactions involving the identified hub genes. In the predicted regulatory network, STAT1 exhibited a high degree among TFs. Signal transducer and activator of transcription 1 (STAT1), an interferon‐responsive transcription factor, has been implicated in both SLE [72] and periodontitis [73]. Among miRNAs, miR‐146a also showed a high degree. miR‐146a levels are higher in patients with aggressive periodontitis than in healthy controls, and it may serve as an indicator of periodontal disease severity [74]. Additionally, miR‐146a has been reported to alleviate kidney injury in SLE mice by regulating classical and non‐classical NF‐κB signaling pathways [75]. Overall, these computationally predicted TF–miRNA interactions provide hypotheses for potential regulatory mechanisms linking periodontitis and SLE, but their biological relevance requires further experimental validation.

Using the DSigDB database, the five hub genes were used to predict candidate compounds, and the top ten compounds were identified. Among these, PGE2‐related signaling has been implicated in immune regulation in experimental models of SLE [76]. Pyrrolidine dithiocarbamate (PDTC), an inhibitor of NF‐κB activation, has been reported to attenuate LPS‐induced inflammatory responses and periodontal inflammation [77]. PDTC has also been reported to alleviate renal inflammation in experimental SLE, at least partially through the NF‐κB and MAPK signaling pathways [78]. Capsaicin reduces LPS‐induced TNF‐α and IL‐6 levels in macrophages and lowers intracellular ROS, suggesting potential anti‐inflammatory effects relevant to periodontal disease [79]. Thus, these computational predictions provide candidate compounds for further investigation; however, their potential therapeutic relevance to periodontitis and SLE requires validation in appropriate in vitro and in vivo models.

ROC curve analysis demonstrated varying discriminatory performance of the five hub genes across the training and validation datasets. LY96 showed relatively consistent performance in both periodontitis datasets, while FOS, LY96, and PTGS2 exhibited relatively higher discriminatory performance in the independent SLE validation dataset. In contrast, CCL3 and MMP9 showed less consistent performance across the SLE datasets, indicating that their potential diagnostic relevance should be interpreted cautiously. From a clinical perspective, these findings highlight the potential value of closer periodontal assessment in patients with SLE and greater interdisciplinary awareness of the association between periodontitis and SLE. However, given the exploratory nature of this study and the variability across independent datasets, these candidate genes should not yet be regarded as established clinical biomarkers. Further validation in larger, independent, and clinically well‐characterized cohorts is required before their potential diagnostic or risk‐stratification value can be determined.

Nevertheless, this study has several limitations. First, although the hub genes were validated by qPCR in a clinical cohort (*n* = 15 per group), larger, independent, and multicenter cohorts are needed to establish their robustness and clinical relevance. Second, differences in biological sources and cellular composition between the periodontitis and SLE datasets may have influenced the identification of shared molecular and immune signatures. Third, heterogeneity in study populations, disease severity, treatment status, and experimental platforms may have affected the results, and not all candidate genes were consistently validated across independent datasets.

Future studies should validate these candidate genes in larger, independent, and clinically well‐characterized cohorts and further investigate their functional roles in the potential shared mechanisms of periodontitis and SLE using in vitro and in vivo models. In addition, experimental validation of the predicted regulatory networks and candidate compounds will be necessary to determine their biological relevance and potential translational value.

## 5 Conclusion

In conclusion, our integrative bioinformatics and clinical validation analyses identified CCL3, LY96, FOS, MMP9, and PTGS2 as candidate genes associated with periodontitis and SLE. FOS, LY96, and PTGS2 showed consistent expression patterns across the analyzed datasets, whereas CCL3 and MMP9 exhibited dataset‐dependent variation in SLE. Clinical qPCR validation showed significantly increased expression of all five hub genes in both disease cohorts. These findings provide insight into the shared molecular features of periodontitis and SLE, although further validation in larger, independent cohorts is required.

## Author Contributions

**Yafang Li:** data curation, validation, investigation, writing – original draft, resources, methodology. **Xin Xu:** investigation, formal analysis. **Zhijing Wu:** investigation, software, data curation, conceptualization. **Feng Li:** software, supervision. **Jie Zhang:** investigation, writing – review and editing, funding acquisition, visualization, project administration.

## Ethics Statement

This study was approved by the Ethics Committee of the First Affiliated Hospital of Nanchang University (Approval No. IITS2024753) and was conducted in accordance with the Declaration of Helsinki. All participants provided written informed consent prior to participation.

## Conflicts of Interest

The authors declare no conflicts of interest.

## Supporting information


Supporting File 1

Supporting File 2

## Data Availability

Further information on the data set and materials is available from the corresponding author upon reasonable request.
