# Supplementary material for: Identification and Validation of Hub Genes Associated With Periodontitis and Systemic Lupus Erythematosus
Source: Immun Inflamm Dis. 2026 Nov 5;14(11):e70588. doi: 10.1002/iid3.70588 (PMC13737472; doi:10.1002/iid3.70588)
Supplement: Supplementary file 1 [file IID3-14-e70588-s001.docx]

**Supplementary Table S1**. Demographic and clinical characteristics of participants included in the clinical validation cohort

| **A. Periodontitis cohort** | | | |
| --- | --- | --- | --- |
| **Characteristic** | **Periodontitis (n = 15)** | **Healthy controls (n = 15)** | ***P* value** |
| Age, years | 36.93 ± 8.03 | 37.27 ± 7.77 | 0.909 |
| Sex, n (%) |  |  |  |
| Female | 8 (53.3) | 8 (53.3) |  |
| Male | 7 (46.7) | 7 (46.7) |  |
| Probing depth (PD), mm | 6.03 ± 0.66 | 2.28 ± 0.29 | < 0.001 |
| Clinical attachment loss (CAL), mm | 4.63 ± 1.01 | 0.00 ± 0.00 | < 0.001 |
| Bleeding on probing (BOP), % | 63.81 ± 11.05 | 3.59 ± 2.95 | < 0.001 |

| **B. SLE cohort** | | | |
| --- | --- | --- | --- |
| **Characteristic** | **SLE (n = 15)** | **Healthy controls (n = 15)** | ***P* value** |
| Age, years | 31.87 ± 7.55 | 33.53 ± 9.46 | 0.598 |
| Sex, n (%) |  |  | 1.000 |
| Female | 13 (86.7) | 13 (86.7) |  |
| Male | 2 (13.3) | 2 (13.3) |  |
| SLEDAI score | 9.73 ± 2.84 | — | — |
| Disease duration, years | 5.80 (3.95–6.85) | — | — |
| Treatment-naïve, n (%) | 15 (100) | — | — |

Data are presented as mean ± standard deviation (SD), median (interquartile range), or n (%), as appropriate. Age was compared using the independent two-sample Student’s t-test, and sex was compared using Fisher’s exact test. Periodontal clinical parameters were compared using the Mann–Whitney U test. PD, probing depth; CAL, clinical attachment loss; BOP, bleeding on probing; SLE, systemic lupus erythematosus; SLEDAI, Systemic Lupus Erythematosus Disease Activity Index.
